# Supplementary material for: Should Peak Dose Be Used to Prescribe Spatially Fractionated Radiation Therapy?—A Review of Preclinical Studies
Source: Cancers (Basel). 2022 Jul 26;14(15):3625. doi: 10.3390/cancers14153625 (PMC9330631; doi:10.3390/cancers14153625)
Supplement: Supplementary file 1 [file cancers-14-03625-s001.zip › Supplementary Figures.pdf]

Index of Correlation Matrices:

1. ALL (MRT + MBRT) ________ 1

2. MRT ________ 2

3. MBRT ________ 3

4. MRT (brain Tumors only) ________ 4

5. MBRT (brain tumors only) ________ 5

6. Proton MBRT ________ 6

7. Photon MBRT ________ 7

1. ALL (MRT + MBRT)

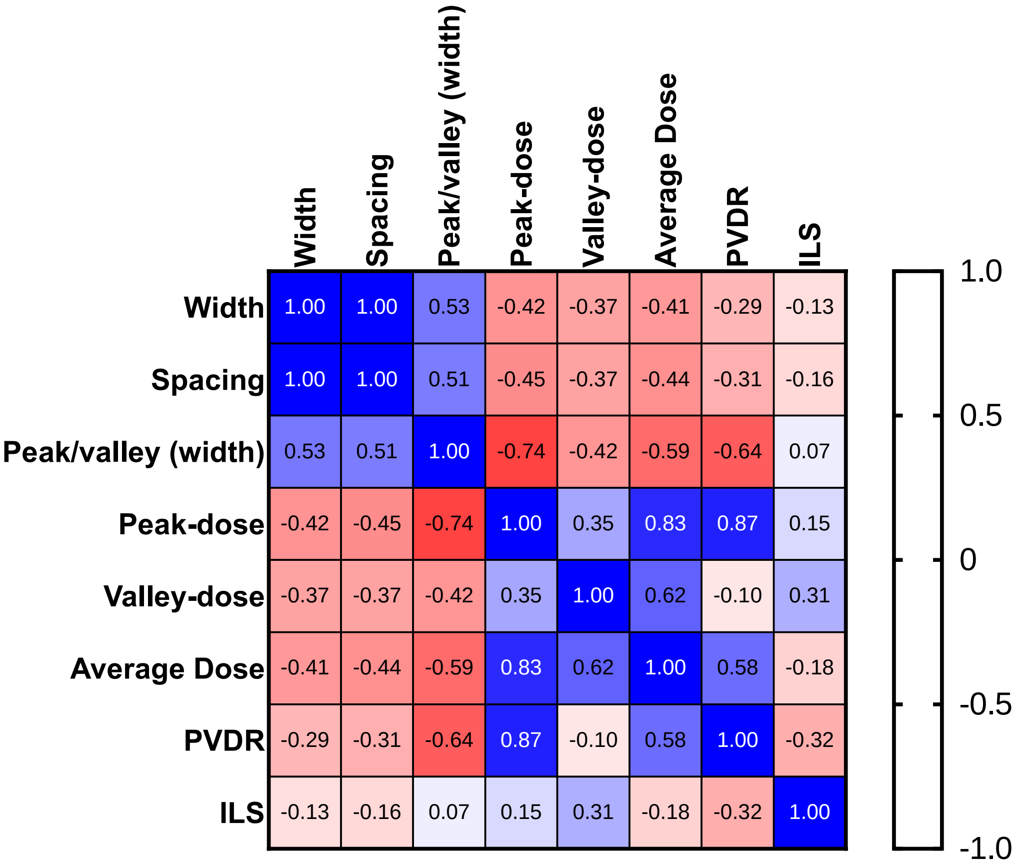

Correlation matrix of ALL (MRT+MBRT)

2. MRT

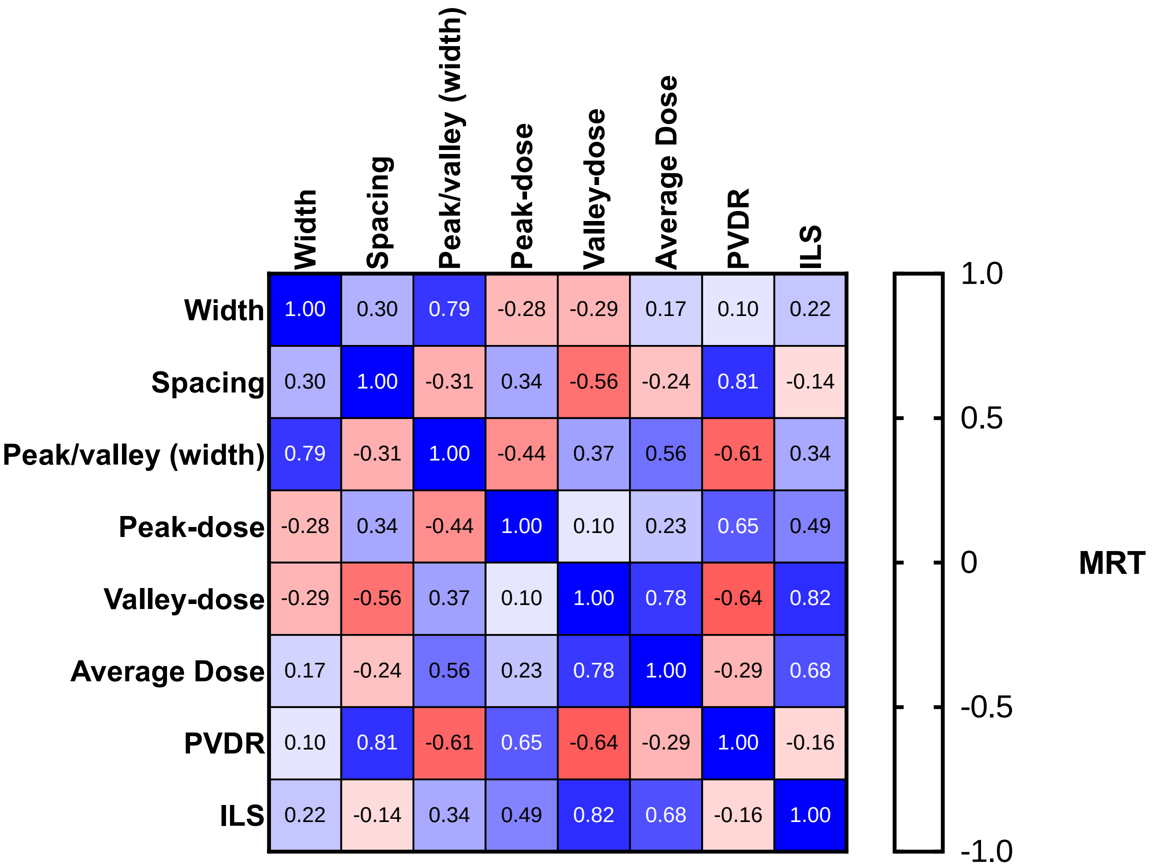

Correlation matrix of MRT

3. MBRT (protons and photons)

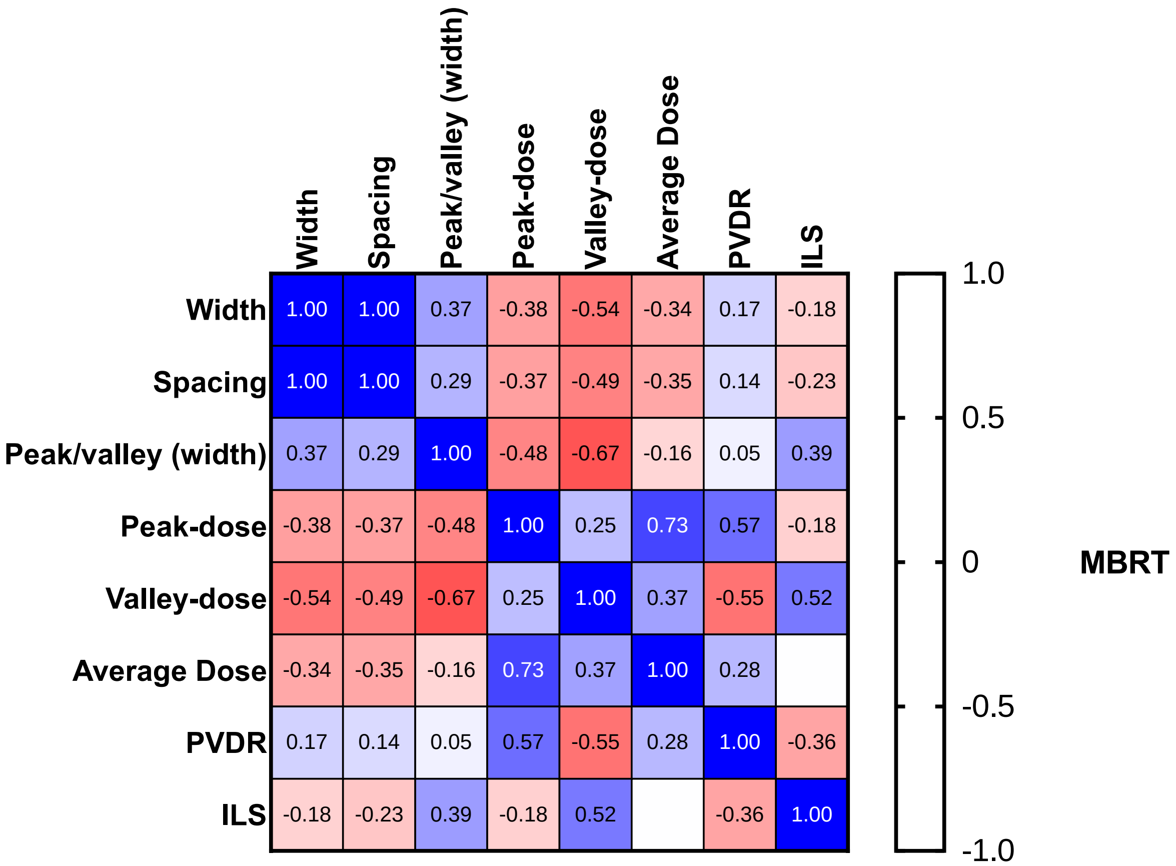

Correlation matrix of MBRT (proton & photons)

4. MRT (brain Tumors only)

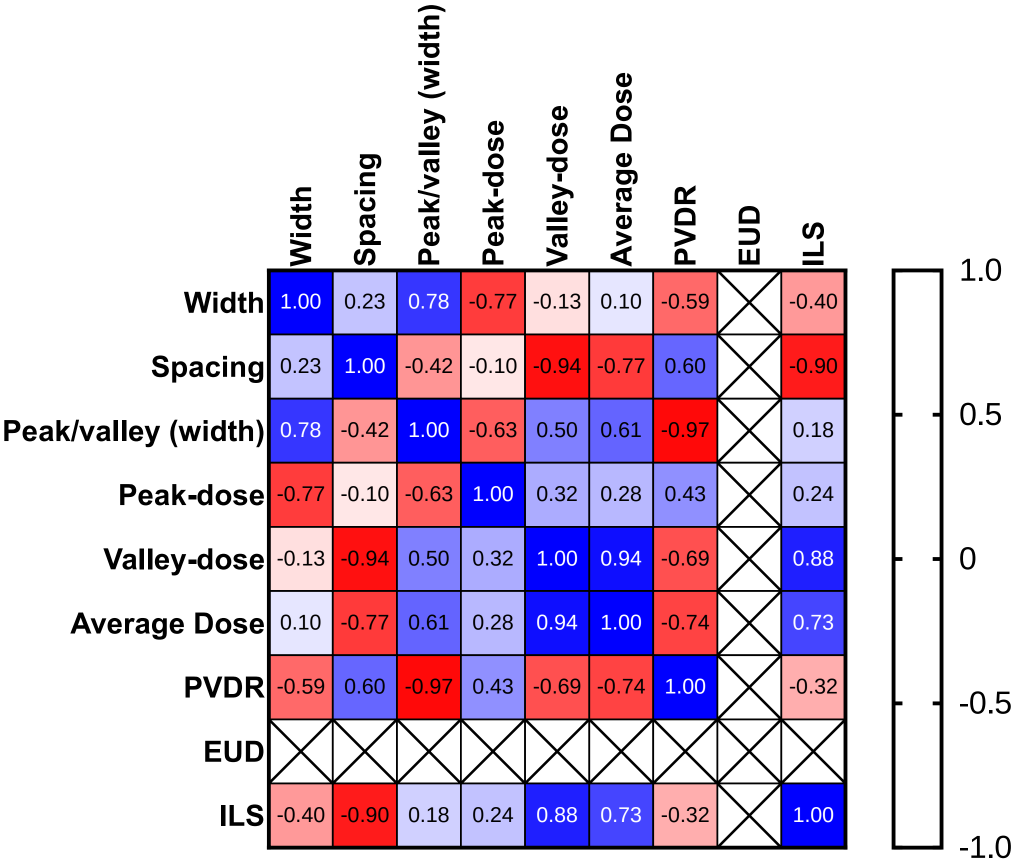

Correlation matrix of brain tumors in MRT

5. MBRT (brain tumors only)

	Width	Spacing	Peak/valley (width)	Peak-dose	Valley-dose	Average Dose	PVDR	EUD	ILS
**Width**	1.00	1.00	-0.98	-0.69	0.67	-0.53	-0.90		-0.63
**Spacing**	1.00	1.00	-0.99	-0.65	0.71	-0.46	-0.92		-0.63
**Peak/valley (width)**	-0.98	-0.99	1.00	0.64	-0.78	0.40	0.95		0.64
**Peak-dose**	-0.69	-0.65	0.64	1.00	-0.75	0.69	0.83		-0.60
**Valley-dose**	0.67	0.71	-0.78	-0.75	1.00	-0.14	-0.91		0.56
**Average Dose**	-0.53	-0.46	0.40	0.69	-0.14	1.00	0.38		-0.13
**PVDR**	-0.90	-0.92	0.95	0.83	-0.91	0.38	1.00		-0.33
**EUD**									
**ILS**	-0.63	-0.63	0.64	-0.60	0.56	-0.13	-0.33		1.00

Scale: 1.0, 0.5, 0, -0.5, -1.0

Correlation matrix of Brain tumors in MBRT (protons and photons)

6. Proton MBRT

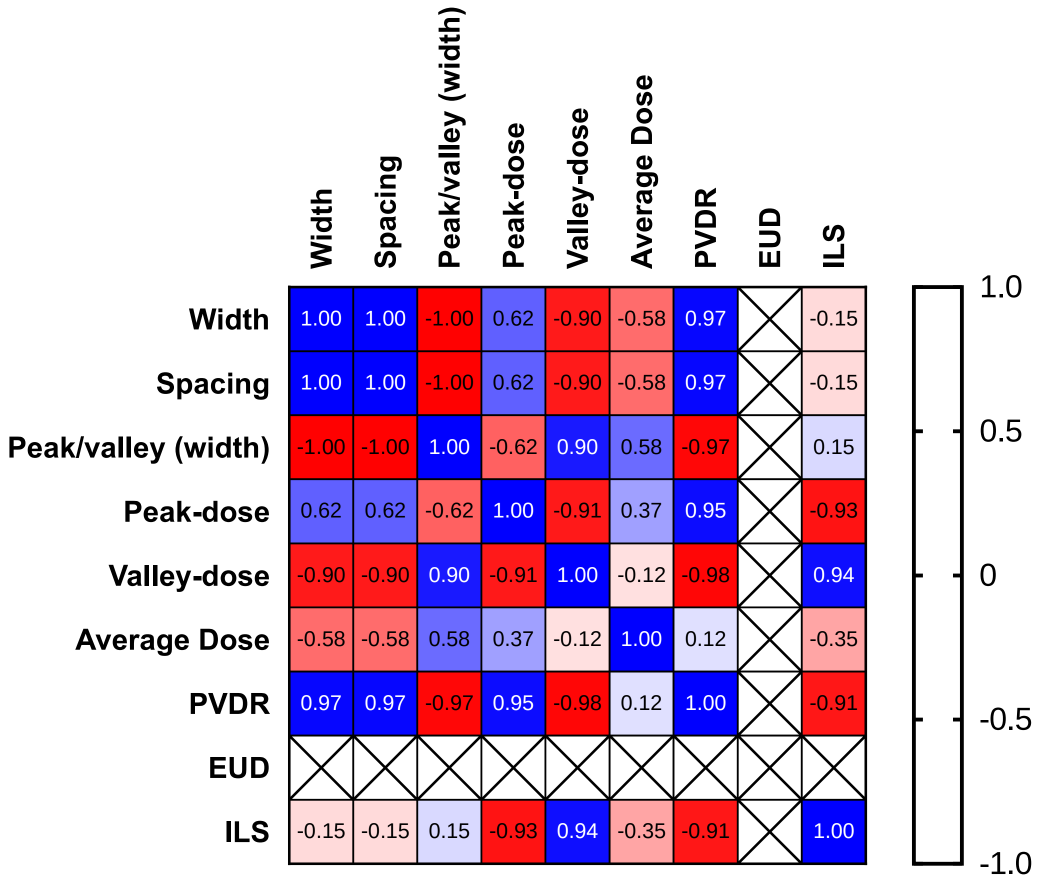

Correlation matrix of proton MBRT

7. Photon MBRT

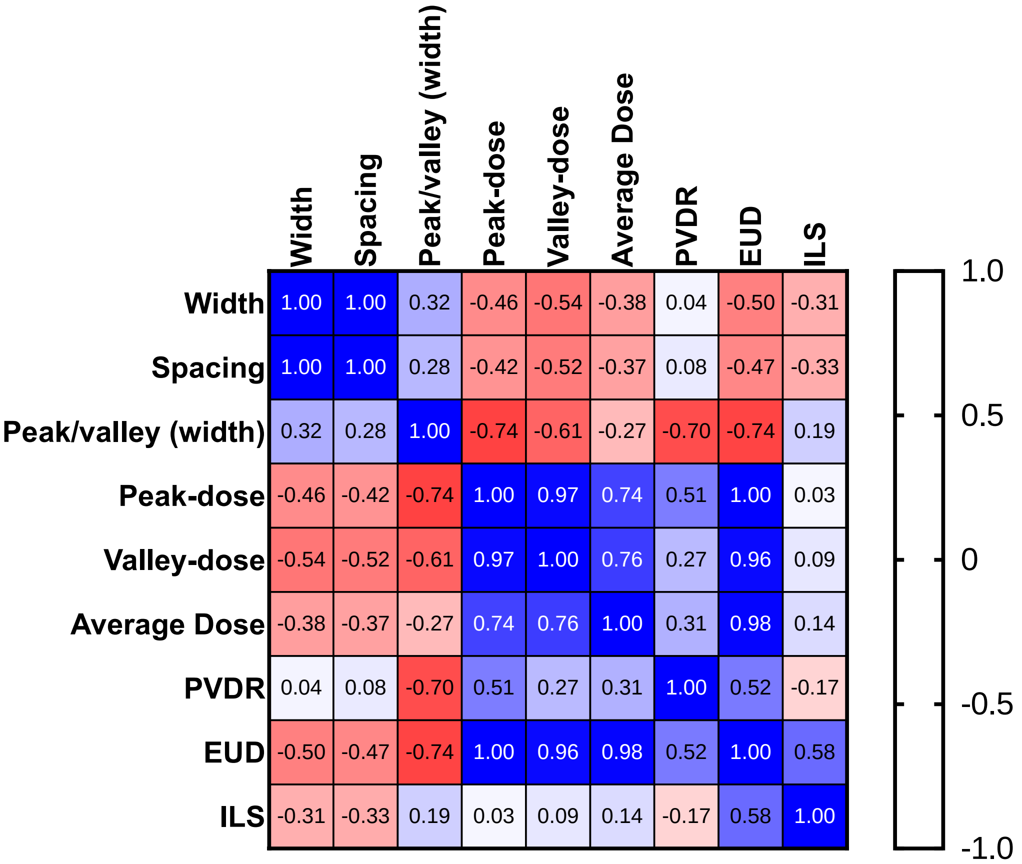

Correlation matrix of Photon MBRT
